# Associations between *ERAP1* Gene Polymorphisms and Psoriasis Susceptibility: A Meta-Analysis of Case-Control Studies

**DOI:** 10.1155/2021/5515868

**Published:** 2021-08-02

**Authors:** Xiujuan Wu, Zongfeng Zhao

**Affiliations:** ^1^Department of Dermatology, Shanghai Xuhui Center Hospital, Shanghai 200031, China; ^2^Department of Scientific Research, Shanghai Xuhui Center Hospital, Shanghai 200031, China

## Abstract

This study is to investigate the relationship of endoplasmic reticulum aminopeptidase 1 (*ERAP1*) gene polymorphisms with psoriasis. Five databases of PubMed, China National Knowledge Infrastructure (CNKI), Embase, Web of Science, and Cochrane Library were searched for potential studies until 25 December 2019. Newcastle-Ottawa Scale (NOS) was used to evaluate the quality of included studies. Meta-analysis was performed with PRISMA. A total of 9 case-control studies including 4858 psoriasis cases and 10,542 healthy controls were included. Three loci of *ERAP1* gene polymorphisms (rs26653, rs30187, and rs27524) were evaluated in this meta-analysis. There was no significant association between rs26653 polymorphism and risk of psoriasis (C vs. G, OR = 1.01, 95% CI: 0.80-1.28, *P* = 0.93). However, there was a significant association between rs30187 polymorphisms and psoriasis susceptibility (T vs. C, OR = 1.23, 95% CI: 1.15-1.32, *P* < 0.00001) and a significant association between rs27524 polymorphisms and psoriasis susceptibility (A vs. G, OR = 1.17, 95% CI: 1.09-1.25, *P* < 0.00001). For there were insufficient data of rs27044, rs151823, rs2248374, and rs2910686, we only conducted a systematic review for them. The rs30187 (C/T) and rs27524 (G/A) polymorphisms of *ERAP1* are associated with increased risk of psoriasis. However, no significant association is observed between rs26653 (G/C) polymorphism and risk of psoriasis.

## 1. Introduction

Psoriasis is a common chronic inflammatory disorder [1–3]. Psoriasis affects about 2%–3% of the population worldwide, and it is more prevalent in Western countries [4]. There are five types of psoriasis identified up to now, including plaque psoriasis, eruptive psoriasis, inverse psoriasis, pustular psoriasis, and erythrodermic psoriasis [1]. As a kind of skin disease, psoriasis brings physical and psychological burdens to patients. Therefore, it is urgent to improve the quality of life for patients.

**Figure 1:**
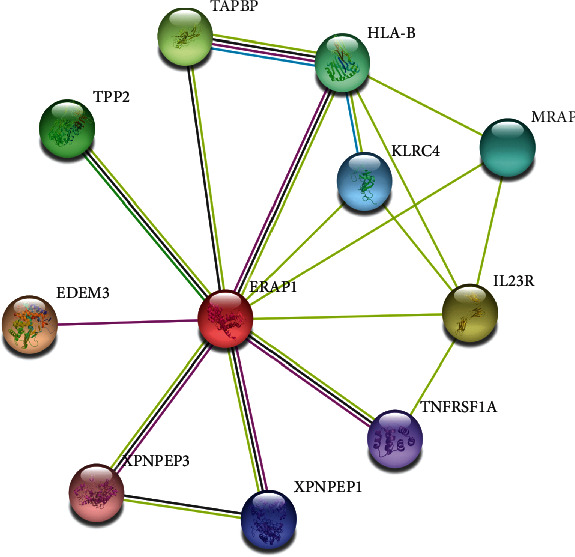
Network of *ERAP1* and its closest functional partners. These data were from the Search Tool for the Retrieval of Interacting Genes (STRING) database (http://string-db.org/).

Many environmental factors have been reported to be associated with psoriasis, such as direct skin trauma, streptococcal throat infection, and human immunodeficiency virus infection [3]. In recent decades, powerful genome-wide association studies and many other candidate gene approaches have identified numerous genes associated with psoriasis risk [5]. Endoplasmic reticulum aminopeptidase 1 (*ERAP1*), which belongs to the M1 family of aminopeptidases, plays a central role as a “molecular ruler,” proteolyzing of N-terminal of the antigenic peptides before their loading onto HLA-I molecules for antigen presentation in the endoplasmic reticulum [6]. *ERAP1* belongs to the oxytocinase subgroup of M1 zinc metallopeptidases, shares 49% sequence similarity, and can form heterodimers [7]. The human *ERAP1* genes are encoded in the short arm chromosome 5q15 in a 167Kb region in the opposite direction, and probably, they have two shared regulatory elements [8]. Polymorphisms in the *ERAP1* gene may affect susceptibility to psoriasis [9]. The rs26653, rs30187, and rs27524 polymorphisms are considered as the three most important loci of *ERAP1* [10, 11]. One genome-wide association study confirmed that the polymorphism of *ERAP1* rs26653 and rs27044 was associated with psoriasis in Chinese patients [12]. rs27524 polymorphism of *ERAP1* is closely related to the susceptibility of psoriasis in Caucasians [13]. rs26653 and rs30187 are associated with early onset psoriasis in Caucasians [14]. Therefore, the polymorphism of *ERAP1* rs27524, rs30187, rs26653, and rs27044 may play significant roles in patients with psoriasis. *ERAP1* is upexpressed by TNF-*α* and IFN-*γ* stimulation, indicating its major function in antigen-presenting machinery [15]. *ERAP1* plays a significant role in the migration and proliferation of endothelial cells as a vital factor for vessel regeneration [16]. The interrelation network of *ERAP1* and its nearest associated functional protein partners are illustrated in Figure 1.

Herein, considering the limited sample sizes and poor statistical power of each individual study, we conducted this meta-analysis. Our findings may provide a more comprehensive evaluation on the association of *ERAP1* polymorphisms with psoriasis susceptibility.

## 2. Materials and Methods

### 2.1. Search Strategy

The online databases of PubMed, China National Knowledge Infrastructure (CNKI), Embase, Web of Science, and Cochrane Library were searched for eligible studies until 25 December 2019. Medical Subject Headings (MeSH) together with free terms were as keywords for literature searching. The following searching strategies for PubMed were employed: (polymorphism OR single nucleotide polymorphism OR variation OR gene) AND (psoriasis OR “Psoriasis” [17]) AND (“*ERAP1* protein, human” [Supplementary Concept] OR *ERAP1* OR Adipocyte-derived leucine aminopeptidase, human OR Type 1 tumor necrosis factor receptor shedding aminopeptidase regulator, human OR endoplasmic-reticulum aminopeptidase-1, human).

### 2.2. Inclusion and Exclusion Criteria

Studies in accordance with the following criteria were included: case-control studies with subjects of human; studies with enough original data to calculate odds ratios (ORs); studies with psoriasis diagnosed according to clinical diagnosis criteria; studies on the relationship between *ERAP1* polymorphisms and psoriasis predisposition. Accordingly, studies that met the following criteria were excluded: reviews, conference abstracts, case reports, ascertained alleles, and overlapping data.

### 2.3. Quality Assessment

The methodological quality of each eligible study was evaluated by two investigators, respectively, in compliance with Newcastle-Ottawa Scale (NOS). NOS contains three aspects: selection (four items), comparability (two items), and the outcomes of case-control studies (three items). Each included study was scored as 0-9 points according to these items. A higher score indicated a better quality. Studies with scores ≥ 5 points were considered to have a high quality for further analysis. When disagreements occurred between the two investigators, a third reviewer was consulted to make the final decision.

### 2.4. Data Extraction

The following information was extracted from each study by two independent investigators: first author, year of publication, ethnicity of study population, numbers of cases and controls, and the allele frequencies of the *ERAP1* polymorphisms, Hardy-Weinberg equilibrium (HWE) results.

### 2.5. Statistical Analysis

This meta-analysis was performed with the Preferred Reporting Items for Systematic Review and Meta-Analyses (PRISMA) [18]. The allele model of inheritance was used to analyze the data due to a lack of sufficient information. The association between *ERAP1* gene polymorphisms and psoriasis was calculated by merging ORs with 95% CIs (confidence intervals). The *Q*-statistical test and *I*^2^ test were employed to evaluate the heterogeneity among all included studies [19]. The random-effects model was used to combine the data in the cases of heterogeneity (*P* < 0.1 or *I*^2^ > 50%), or the fixed-effects model was used when it was out of heterogeneity (*P* > 0.1 or *I*^2^ < 50%) [20, 21]. A leave-one-out sensitivity analysis was used to explore the stability of pooled results by omitting each investigated study at a time. A funnel plot of publication bias and forest plots of meta-analysis were plotted with Review Manager Version 5.3 software (Cochrane Collaboration, Software Update, Oxford, United Kingdom).

## 3. Results

### 3.1. Literature Search

**Figure 2:**
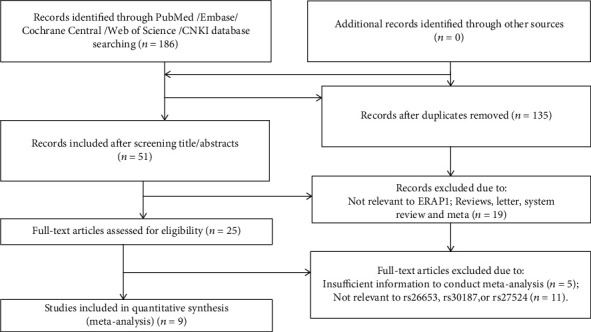
Flow diagram of literature search and screening.

The detailed process of the literature screening is shown in Figure 2. The primary searching in five databases yielded 186 records, consisting of 49 from PubMed, 41 from Embase, 0 from Cochrane Library, 65 from Web of Science, and 31 from CNKI. After removing 135 duplicate articles, the remaining 51 articles were subjected to screening by title and abstracts; after removing 7 articles irrelevant to ERAP1 and 19 reviews and letters, there were 25 articles that were eligible for full-text review; after excluding 5 article studies with insufficient data and 11 articles with irrelevant data, 9 original case-control studies consisting of 4858 psoriasis cases and 10542 healthy controls were finally included.

### 3.2. Study Characteristics

General characterizations of all included studies are demonstrated in Table 1. All the included studies were published in English from 2011 to 2018. Among them, two of them were from China [22, 23], another two were from Poland [24, 25], the rest were from India [26], UK [27], Netherlands [28], Sweden [14], and Romania [29]. The quality of included studies was evaluated with NOS. The results showed that each included study had a score of more than 5 points (Table 2), indicating good quality of included studies.

### 3.3. Meta-Analysis Results

Due to the limited number of studies on other loci, we only meta-analyzed three loci of rs26653 (G/C), rs30187 (C/T), and rs27524 (G/A) of *ERAP1* polymorphisms.

### 3.4. Relationship between rs26653 (G/C) Polymorphism and Psoriasis Susceptibility

**Figure 3:**
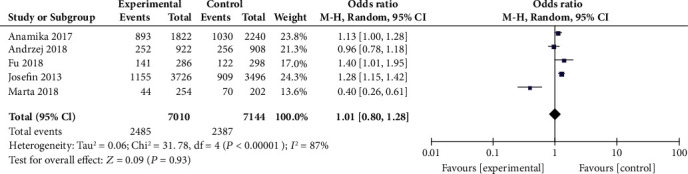
Forest plot of rs26653 in the ERAP1 gene and risk of psoriasis.

Meta-analysis of the relationship between rs26653 polymorphism and psoriasis susceptibility was conducted based on 5 studies consisting of 3505 cases and 3572 controls [14, 22, 24–26]. There was an enormous heterogeneity among the five included studies (*P* < 0.00001, *I*^2^ = 87%). Therefore, the random-effects model was used. The results indicated that no significant association was observed between rs26653 polymorphism and risk of psoriasis (C vs. G, OR = 1.01, 95% CI: 0.80-1.28, *P* = 0.93, Figure 3).

### 3.5. Relationship between rs30187 (C/T) Polymorphism and Psoriasis Susceptibility

**Figure 4:**
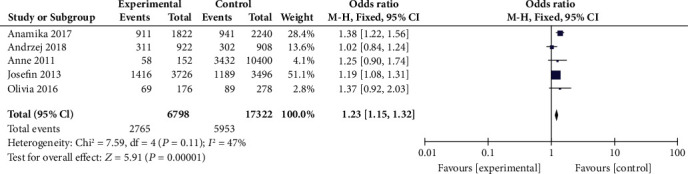
Forest plot of rs30187 in the ERAP1 gene and risk of psoriasis.

Meta-analysis for the relationship between rs30187 (C/T) polymorphism and psoriasis susceptibility was on the basis of five studies [19–21, 23, 24], including 3399 cases and 8661 healthy controls. A marginal heterogeneity was observed among the five included studies (*P* = 0.11, *I*^2^ = 47%), and therefore, the fixed-effects model was used. The results suggested that there was a significant association between rs30187 polymorphisms and psoriasis susceptibility (T vs. C, OR = 1.23, 95% CI: 1.15-1.32, *P* < 0.00001, Figure 4).

### 3.6. Relationship between rs27524 (G/A) Polymorphism and Psoriasis Susceptibility

**Figure 5:**
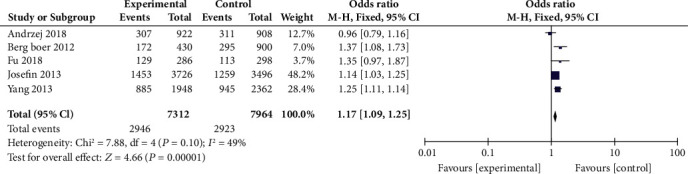
Forest plot of rs27524 in the *ERAP1* gene and risk of psoriasis.

Analysis of the association of rs27524 polymorphism and psoriasis susceptibility was also based on five studies [14, 22, 23, 25, 28], including a total of 3656 psoriasis cases and 3982 healthy controls. There was a marginal heterogeneity among the five included studies, and thus, the fixed-effects model was used. The results displayed that there was a significant association between rs27524 polymorphisms and psoriasis susceptibility (A vs. G, OR = 1.17, 95% CI: 1.09-1.25, *P* < 0.00001, Figure 5).

### 3.7. Relationship between Other *ERAP* Gene Polymorphisms and Psoriasis Susceptibility

Since there were only a few studies that reported detailed information on rs27044, rs151823, rs2248374, and rs2910686, we only performed a narrative description for these loci. Three studies reported rs27044 polymorphisms in the *ERAP1* gene and psoriasis susceptibility. In Wiśniewski et al.'s study [25], rs27044 was not associated with psoriasis in the Polish population (G vs. C, OR = 1.05, 95% CI: 0.86-1.29), which was consistent with the results by Popa et al. [29] (G vs. C, OR = 1.40, 95% CI:0.92, 2.12). However, rs27044 polymorphism was found to be significantly associated with psoriasis risk in Das et al.'s study [26] (G vs. C, OR = 1.24, 95% CI: 1.07–1.43).

Two studies [30, 31] reported that rs151823 gene polymorphisms of *ERAP1* may be also involved in the pathogenesis of psoriasis and treatment response of biologic therapies to psoriasis.

The rs2248374 and rs2910686 are two loci located in *ERAP2*, which plays the same roles as *ERAP1* in biological process. In Popa et al.'s study [29], rs2248374 polymorphism was found to be significantly associated with psoriasis risk (A vs. G, OR = 1.45, 95% CI: 1.01-2.10) while rs2910686 polymorphism was not associated with psoriasis risk (C vs. T, OR = 1.41, 95% CI: 0.97-2.05).

### 3.8. Sensitivity Analysis and Publication Bias

**Figure 6:**
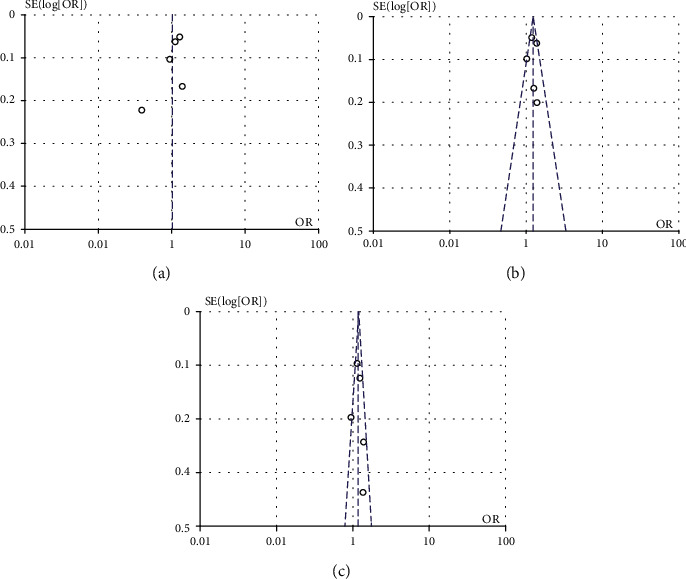
Analysis of publication bias. Funnel plot of included studies on rs26653 (a), rs30187 (b), and rs27524 (c) of the *ERAP1* gene.

A leave-one-out sensitivity analysis was used to explore the stability of pooled results. The overall effect size did not change after omitting one included study at a time. Publication bias detection was made by visual inspection of the funnel plots. The results did not identify substantial asymmetry (Figure 6), indicating no publication bias.

## 4. Discussion

*ERAP*1 was identified by GWAS to be preferentially associated with early onset psoriasis among Chinese [12]. *ERAP1* polymorphism was first reported to be associated with the disease of ankylosing spondylitis in 2007 [32]. After that, the polymorphisms in *ERAP1* have also been found to be associated with Behcet's disease by genome-wide association studies [13, 33, 34], and since then, replication studies were performed by researchers all over the world. Many polymorphisms in the *ERAP1* gene have been shown to be associated with genetic susceptibility to psoriasis [14]. *ERAP1* had genetic heterogeneity in a Swedish cohort with early onset ankylosing spondylitis [35]. *ERAP1* SNPs are identified as intronic variants in most studies. *ERAP1* haplotype is predisposing to psoriasis in East Asians [8]. *ERAP2* was established upon controlling for the contribution of *ERAP1* in psoriasis [36]. The effect of *ERAP2* on disease susceptibility is often masked by *ERAP1* because of *ERAP1* and *ERAP2* within the same haplotype [37].

High *ERAP1* expression is a risk factor for psoriasis [38]. To the best of our knowledge, the present study is the first and most comprehensive meta-analysis on the association of *ERAP1* gene polymorphisms with psoriasis risk. Of the 9 included studies, only Asian and Caucasian population was eligible. Since there was not enough information on the exact number for all genotypes of every individual involved in the original study, we only analyzed the data using an allele model. The meta-analysis results indicated that carriers with the minor allele of rs30187 and rs27524 had an increased risk of psoriasis, while carriers with the minor allele of rs26653 seemed to have neither increased nor decreased risk of psoriasis. Subgroup analyses were not conducted due to a lack of enough original studies.

The role of ERAP1 variations is underlined by the association of rs27524 in HLA-Cw6 positive individuals [13, 39]. Studies [40, 41] have shown that rs26653 is associated with a risk of psoriasis independent of hla-c06 expression. rs26653 SNP may be involved in psoriasis by targeting important components such as IL-17/IL-23 [14]. rs26653 (R127 P) in the junction domains could indirectly affect either enzymatic activity or specificity by switching between open and close conformations [14]. rs27044 SNP is the most common SNP in almost all populations [33]. It confers a protective effect due to a significant reduction in aminopeptidase activity [42]. Five studies reporting rs26653 polymorphism showed no significant association with psoriasis susceptibility [14, 22, 24–26]. In addition, five studies identified significant association of rs30187 (C/T) polymorphism with psoriasis [14, 25–27, 29]. The five studies reporting rs27524 polymorphism demonstrated a significant susceptibility to psoriasis [14, 22, 23, 25, 28]. Other studies have shown that rs27044 [25, 26, 29] and rs151823 [30, 31] are associated with susceptibility to psoriasis. The rs2248374 and rs2910686 are two loci located in *ERAP2*, which plays the same roles as *ERAP1* [29].

*ERAP1* together with *ERAP2* belongs to the oxytocinase subfamily of M1 zinc metallopeptidases [43] and contains 20 exons and 19 introns [44]. *ERAP1* participates in the final processing of major histocompatibility complex (MHC) class I ligands in the endoplasmatic reticulum by trimming the peptides to a required length for presentation [45, 46]. Apart from the antigen processing pathway of MHC-I molecules, *ERAP1* is also involved in the indirect presentation of cytoplasmic MHC-II ligands [47, 48]. The downregulated expression level of *ERAP1* will upregulate the antigen-presenting properties and deregulate the immunological effect produced by MHC-I molecules in the host defense against pathogens [49–51]. *ERAP1* also participates in the process of oncogenesis [52]. The effect of *ERAP1* on angiotensin II modulates angiogenesis and therefore leads to the occurrence of endometrial carcinoma [52]. *ERAP1* regulates the immune defense against tumorigenesis by affecting the balance of activating and inhibitory signals through MHC-I proteins [53]. *ERAP1* also has other biological functions, such as regulating blood pressure and angiogenesis [41, 43].

Several limitations of the present meta-analysis should not be overlooked. First, the study number and the sample size of included studies were relatively small, which might provide a limited conclusion. Second, the heterogeneity between studies was relatively considerable, and thus, the conclusion of this meta-analysis should be quoted with caution. Third, only studies on Caucasian and Asian populations were included. Whether the conclusions are consistent with those on other populations needs further investigation. Fourth, due to the limited number of included studies, meta-regression analysis was not performed. Last, we only searched English and Chinese databases to perform this work. Potentially relevant articles published in other languages may be ignored.

## 5. Conclusions

In summary, *ERAP1* polymorphism of rs30187 (C/T) and rs27524 (G/A) was associated with an increased risk of psoriasis. However, no significant association between rs26653 (G/C) polymorphism and psoriasis risk was observed. The results of this study may provide a more comprehensive evaluation of the relationship between *ERAP1* polymorphism and psoriasis susceptibility.

## Figures and Tables

**Table 1:** Main characteristics of included studies.

Study	Year	Country	Ethnicity	Case/control	Case		Control		HWE
					Mutant allele	Wild allele	Mutant allele	Wild allele	
*rs26653 (G/C)*									
Anamika	2017	India	Indian	911/1120	893	929	1030	1210	0.60
Andrzej	2018	Poland	Polish	461/454	252	670	256	652	0.02
Fu	2018	China	Han	143/149	141	145	122	176	>0.05
Josefin	2013	Sweden	Caucasian	1863/1748	1155	2571	909	2587	NA
Marta	2018	Poland	Polish	127/101	44	210	70	132	NA
*rs30187 (C/T)*									
Anamika	2017	India	Indian	911/1120	911	911	941	1299	0.59
Andrzej	2018	Poland	Polish	461/454	311	611	302	606	0.67
Anne	2011	UK	Caucasian	76/5200	58	94	3432	6968	>0.05
Josefin	2013	Sweden	Caucasian	1863/1748	1416	2310	1189	2307	NA
Olivia	2016	Romania	Caucasian	88/139	69	107	89	189	>0.05
*rs27524 (G/A)*									
Andrzej	2018	Poland	Polish	461/454	307	615	311	597	0.83
Bergboer	2012	Netherlands	Caucasian	215/450	172	258	295	605	>0.05
Fu	2018	China	Han	143/149	129	157	113	185	>0.05
Josefin	2013	Sweden	Caucasian	1863/1748	1453	2273	1259	2237	NA
Yang	2013	China	Han	974/1181	885	1063	945	1417	0.58

Note: HWE: Hardy-Weinberg equilibrium.

**Table 2:** Quality assessment of included studies according to the Newcastle-Ottawa Scale.

Item/study	Adequate definition of cases	Representativeness of cases	Selection of control subjects	Definition of control subjects	Control for important factor or additional factor	Exposure assessment	Same method of ascertainment for all subjects	Nonresponse rate	Total score
Anamika 2017	1	0	0	1	1	1	1	1	6
Andrzej 2018	1	0	1	1	1	1	1	1	7
Anne 2011	1	0	0	1	1	1	1	1	6
Bergboer 2012	1	0	0	1	1	1	1	1	6
Fu 2018	1	0	0	1	1	1	1	1	6
Josefin 2013	1	0	0	1	1	1	1	1	6
Marta 2018	1	0	0	1	1	1	1	1	6
Olivia 2016	1	0	0	1	1	1	1	1	6
Yang 2013	1	0	1	1	1	1	1	1	7

## Data Availability

Data are available from the corresponding author.

## References

[1] Boehncke W. H., Schön M. P. (2015). Psoriasis. *Lancet (London, England)*.

[2] Di Meglio P., Villanova F., Nestle F. O. (2014). Psoriasis. *Cold Spring Harbor perspectives in medicine*.

[3] Weigle N., McBane S. (2013). Psoriasis. *American family physician*.

[4] Perera G. K., Di Meglio P., Nestle F. O. (2012). Psoriasis. *Annual review of pathology*.

[5] Mahil S. K., Capon F., Barker J. N. (2015). Genetics of psoriasis. *Dermatologic Clinics*.

[6] Babaie F., Hosseinzadeh R., Ebrazeh M. (2020). The roles of ERAP1 and ERAP2 in autoimmunity and cancer immunity: new insights and perspective. *Molecular Immunology*.

[7] Vitulano C., Tedeschi V., Paladini F., Sorrentino R., Fiorillo M. T. (2017). The interplay between HLA-B27 and ERAP1/ERAP2 aminopeptidases: from anti-viral protection to spondyloarthritis. *Clinical & Experimental Immunology*.

[8] Sun L. D., Cheng H., Wang Z. X. (2010). Association analyses identify six new psoriasis susceptibility loci in the Chinese population. *Nature genetics*.

[9] Sukhov A., Adamopoulos I. E., Maverakis E. (2016). Interactions of the immune system with skin and bone tissue in psoriatic arthritis: a comprehensive review. *Clinical reviews in allergy & immunology*.

[10] Yao Y., Liu N., Zhou Z., Shi L. (2019). Influence of ERAP1 and ERAP2 gene polymorphisms on disease susceptibility in different populations. *Human immunology*.

[11] Reeves E., James E. (2018). The role of polymorphic ERAP1 in autoinflammatory disease. *Bioscience reports*.

[12] Tang H., Jin X., Li Y. (2014). A large-scale screen for coding variants predisposing to psoriasis. *Nature genetics*.

[13] Genetic Analysis of Psoriasis Consortium & the Wellcome Trust Case Control Consortium 2, Strange A., Capon F. (2010). A genome-wide association study identifies new psoriasis susceptibility loci and an interaction between *HLA*-*C* and *ERAP1*. *Nature genetics*.

[14] Lysell J., Padyukov L., Kockum I., Nikamo P., Ståhle M. (2013). Genetic association with *ERAP1* in psoriasis is confined to disease onset after puberty and not dependent on *HLA-C*∗*06*. *The Journal of investigative dermatology*.

[15] Forloni M., Albini S., Limongi M. Z. (2010). NF-kappaB, and not MYCN, regulates MHC class I and endoplasmic reticulum aminopeptidases in human neuroblastoma cells. *Cancer research*.

[16] Goto Y., Ogawa K., Hattori A., Tsujimoto M. (2011). Secretion of endoplasmic reticulum aminopeptidase 1 is involved in the activation of macrophages induced by lipopolysaccharide and interferon-*γ*. *The Journal of biological chemistry*.

[17] Ramesh G., Sai N. V. B., Gururaj P., Bhupal R., Patel N. (2018). Association of metabolic syndrome and level of hs-CRP, Lp(a), and serum ferritin in young Asian patients (</=45 years) with acute myocardial infarction. *Interventional Medicine and Applied Science*.

[18] Moher D., Liberati A., Tetzlaff J., Altman D. G., The PRISMA Group (2009). Preferred reporting items for systematic reviews and meta-analyses: the PRISMA statement. *PLoS medicine*.

[19] Higgins J. P., Thompson S. G., Deeks J. J., Altman D. G. (2003). Measuring inconsistency in meta-analyses. *BMJ (Clinical research ed)*.

[20] Mantel N., Haenszel W. (1959). Statistical aspects of the analysis of data from retrospective studies of disease. *Journal of the National Cancer Institute*.

[21] DerSimonian R., Laird N. (1986). Meta-analysis in clinical trials. *Controlled Clinical Trials*.

[22] Fu Y., Li X., Chen Y., Liu R., Wang R., Bai N. (2018). Association of *ERAP1* gene polymorphisms with the susceptibility to psoriasis vulgaris: a case-control study. *Medicine*.

[23] Yang Q., Liu H., Qu L. (2013). Investigation of 20 non-HLA (human leucocyte antigen) psoriasis susceptibility loci in Chinese patients with psoriatic arthritis and psoriasis vulgaris. *The British journal of dermatology*.

[24] Stawczyk-Macieja M., Szczerkowska-Dobosz A., Rębała K. (2018). *ERAP1* and *HLA-C*∗*06* are strongly associated with the risk of psoriasis in the population of northern Poland. *Advances in Dermatology and Allergology/Postȩpy Dermatologii i Alergologii*.

[25] Wiśniewski A., Matusiak Ł., Szczerkowska-Dobosz A., Nowak I., Łuszczek W., Kuśnierczyk P. (2018). The association of ERAP1 and ERAP2 single nucleotide polymorphisms and their haplotypes with psoriasis vulgaris is dependent on the presence or absence of the HLA-C∗06:02 allele and age at disease onset. *Human immunology*.

[26] Das A., Chandra A., Chakraborty J. (2017). Associations of ERAP1 coding variants and domain specific interaction with HLA-C∗06 in the early onset psoriasis patients of India. *Human immunology*.

[27] Hinks A., Martin P., Flynn E. (2011). Subtype specific genetic associations for juvenile idiopathic arthritis: *ERAP1* with the enthesitis related arthritis subtype and *IL23R* with juvenile psoriatic arthritis. *Arthritis research & therapy*.

[28] Bergboer J. G., Oostveen A. M., de Jager M. E. (2012). Paediatric-onset psoriasis is associated withERAP1andIL23Rloci,LCE3C_LCE3Bdeletion and HLA-C∗06. *The British journal of dermatology*.

[29] Popa O. M., Cherciu M., Cherciu L. I. (2016). ERAP1 and ERAP2 gene variations influence the risk of psoriatic arthritis in Romanian population. *Archivum Immunologiae et Therapiae Experimentalis*.

[30] Masouri S., Stefanaki I., Ntritsos G. (2016). A pharmacogenetic study of psoriasis risk variants in a Greek population and prediction of responses to anti-TNF-*α* and anti-IL-12/23 agents. *Molecular diagnosis & therapy*.

[31] Yin X. Y., Zhang R., Cheng H. (2013). Gene–gene interactions between HLA-C, ERAP1, TNFAIP3 and TRAF3IP2 and the risk of psoriasis in the Chinese Han population. *The British journal of dermatology*.

[32] Wellcome Trust Case Control Consortium, Australo-Anglo-American Spondylitis Consortium (TASC), Burton P. R. (2007). Association scan of 14,500 nonsynonymous SNPs in four diseases identifies autoimmunity variants. *Nature genetics*.

[33] Kirino Y., Bertsias G., Ishigatsubo Y. (2013). Genome-wide association analysis identifies new susceptibility loci for Behçet’s disease and epistasis between HLA-B∗51 and ERAP1. *Nature genetics*.

[34] Gül A. (2014). Genetics of Behçet’s disease: lessons learned from genomewide association studies. *Current opinion in rheumatology*.

[35] Montilla C., del Pino-Montes J., Collantes-Estevez E. (2012). Clinical features of late-onset ankylosing spondylitis: comparison with early-onset disease. *The Journal of rheumatology*.

[36] Schittenhelm R. B., Sian T. C., Wilmann P. G., Dudek N. L., Purcell A. W. (2015). Revisiting the arthritogenic peptide theory: quantitative not qualitative changes in the peptide repertoire of HLA-B27 allotypes. *Arthritis & rheumatology (Hoboken, NJ)*.

[37] Yin X., Low H. Q., Wang L. (2015). Genome-wide meta-analysis identifies multiple novel associations and ethnic heterogeneity of psoriasis susceptibility. *Nature communications*.

[38] López de Castro J. A., Alvarez-Navarro C., Brito A., Guasp P., Martín-Esteban A., Sanz-Bravo A. (2016). Molecular and pathogenic effects of endoplasmic reticulum aminopeptidases ERAP1 and ERAP2 in MHC-I-associated inflammatory disorders: towards a unifying view. *Molecular immunology*.

[39] Chen L., Tsai T. F. (2018). *HLA*‐*Cw6* and psoriasis. *British Journal of Dermatology*.

[40] Evnouchidou I., Kamal R. P., Seregin S. S. (2011). Cutting edge: coding single nucleotide polymorphisms of endoplasmic reticulum aminopeptidase 1 can affect antigenic peptide generation in vitro by influencing basic enzymatic properties of the enzyme. *Journal of immunology (Baltimore, Md: 1950)*.

[41] Goto Y., Hattori A., Ishii Y., Tsujimoto M. (2006). Reduced activity of the hypertension-associated Lys528Arg mutant of human adipocyte-derived leucine aminopeptidase (A-LAP)/ER-aminopeptidase-1. *FEBS letters*.

[42] Yao Y., Wiśniewski A., Ma Q. (2016). Single nucleotide polymorphisms of the ERAP1 gene and risk of NSCLC: a comparison of genetically distant populations, Chinese and Caucasian. *Archivum Immunologiae et Therapiae Experimentalis*.

[43] Tsujimoto M., Hattori A. (2005). The oxytocinase subfamily of M1 aminopeptidases. *Biochimica et Biophysica Acta*.

[44] Hattori A., Matsumoto K., Mizutani S., Tsujimoto M. (2001). Genomic organization of the human adipocyte-derived leucine aminopeptidase gene and its relationship to the placental leucine aminopeptidase/oxytocinase gene. *Journal of Biochemistry*.

[45] Saric T., Chang S. C., Hattori A. (2002). An IFN-*γ*-induced aminopeptidase in the ER, ERAP1, trims precursors to MHC class I-presented peptides. *Nature Immunology*.

[46] Serwold T., Gaw S., Shastri N. (2001). ER aminopeptidases generate a unique pool of peptides for MHC class I molecules. *Nature Immunology*.

[47] Dragovic S. M., Hill T., Christianson G. J. (2011). Proteasomes, TAP, and endoplasmic reticulum-associated aminopeptidase associated with antigen processing control CD4^+^ Th cell responses by regulating indirect presentation of MHC class II-restricted cytoplasmic antigens. *Journal of immunology (Baltimore, Md: 1950)*.

[48] Spencer C. T., Dragovic S. M., Conant S. B. (2013). Sculpting MHC class II-restricted self and non-self peptidome by the class I Ag-processing machinery and its impact on Th-cell responses. *European Journal of Immunology*.

[49] Blanchard N., Gonzalez F., Schaeffer M. (2008). Immunodominant, protective response to the parasite *Toxoplasma gondii* requires antigen processing in the endoplasmic reticulum. *Nature Immunology*.

[50] Yan J., Parekh V. V., Mendez-Fernandez Y. (2006). In vivo role of ER-associated peptidase activity in tailoring peptides for presentation by MHC class Ia and class Ib molecules. *The Journal of experimental medicine*.

[51] Firat E., Saveanu L., Aichele P. (2007). The role of endoplasmic reticulum-associated aminopeptidase 1 in immunity to infection and in cross-presentation. *Journal of immunology (Baltimore, Md: 1950)*.

[52] Watanabe Y., Shibata K., Kikkawa F. (2003). Adipocyte-derived leucine aminopeptidase suppresses angiogenesis in human endometrial carcinoma via renin-angiotensin system. *Clinical cancer research: an official journal of the American Association for Cancer Research*.

[53] Alvarez-Navarro C., López de Castro J. A. (2014). ERAP1 structure, function and pathogenetic role in ankylosing spondylitis and other MHC-associated diseases. *Molecular immunology*.

